# Genetic Inhibition Of The Ubiquitin Ligase Rnf5 Attenuates Phenotypes Associated To F508del Cystic Fibrosis Mutation

**DOI:** 10.1038/srep12138

**Published:** 2015-07-17

**Authors:** Valeria Tomati, Elvira Sondo, Andrea Armirotti, Emanuela Caci, Emanuela Pesce, Monica Marini, Ambra Gianotti, Young Ju Jeon, Michele Cilli, Angela Pistorio, Luca Mastracci, Roberto Ravazzolo, Bob Scholte, Ze’ev Ronai, Luis J. V. Galietta, Nicoletta Pedemonte

**Affiliations:** 1 Istituto Giannina Gaslini, Genova, Italy; 2 Department of Drug Discovery and Development, Istituto Italiano di Tecnologia, Genova, Italy; 3 The Burnham Institute for Medical Research, La Jolla, California, USA; 4 IRCCS AOU San Martino–IST, Genova, Italy; 5 Anatomic Pathology Unit, Department of Surgical Sciences and Integrated Diagnostics, University of Genova, Italy; 6 DINOGMI Department, University of Genova, Italy; 7 Cell Biology Department, Erasmus Medical Center, Rotterdam, The Netherlands

## Abstract

Cystic fibrosis (CF) is caused by mutations in the CFTR chloride channel. Deletion of phenylalanine 508 (F508del), the most frequent CF mutation, impairs CFTR trafficking and gating. F508del-CFTR mistrafficking may be corrected by acting directly on mutant CFTR itself or by modulating expression/activity of CFTR-interacting proteins, that may thus represent potential drug targets. To evaluate possible candidates for F508del-CFTR rescue, we screened a siRNA library targeting known CFTR interactors. Our analysis identified RNF5 as a protein whose inhibition promoted significant F508del-CFTR rescue and displayed an additive effect with the investigational drug VX-809. Significantly, RNF5 loss in F508del-CFTR transgenic animals ameliorated intestinal malabsorption and concomitantly led to an increase in CFTR activity in intestinal epithelial cells. In addition, we found that RNF5 is differentially expressed in human bronchial epithelia from CF vs. control patients. Our results identify RNF5 as a target for therapeutic modalities to antagonize mutant CFTR proteins.

Cystic Fibrosis (CF), one of the most common inherited diseases (~1/3000 in Caucasian populations), is caused by mutations in the gene encoding the CF transmembrane conductance regulator (CFTR), a cAMP-regulated chloride channel expressed at the apical membrane of many types of epithelial cells^1^,^2^. In the airways, defective CFTR function alters electrolyte/water transport in several ways. The low chloride transport causes impaired hydration, and reduced bicarbonate secretion affects mucus viscosity, release and detachment^3^,^4^,^5^. Concomitant activity of the epithelial sodium channel (ENaC) drives sodium/fluid absorption thus leading to further dehydration of the epithelial surface. Consequent depletion of the periciliary fluid (PCF) causes the arrest of mucociliary transport^6^, enabling bacteria to colonize airways. Persistence of bacteria in the lung triggers a severe inflammatory response accompanied by progressive loss of respiratory function. Impaired anion transport seen in CF also perturbs function of the pancreas, liver, sweat glands, and reproductive system.

The most common CF mutation, the deletion of phenylalanine 508 (F508del), is responsible for two distinct defects: 1) a processing defect, that is, misfolding of the F508del-CFTR protein, which causes its retention at the endoplasmic reticulum (ER) and premature degradation by the ubiquitin/proteasome system^7^,^8^,^9^,^10^,^11^; and 2) a channel gating defect due to abnormal persistence of a closed state^12^,^13^,^14^,^15^. Several studies have assessed the suitability of F508del-CFTR as a target for pharmacotherapy. Noteworthy, both F508del defects can be rescued *in vitro* using specific small molecules, and thus the defective channel is considered “druggable”. In particular, the gating defect is partially ameliorated by “potentiators”, defined as compounds that acutely stimulate channel activity. Other molecules of interest, known as “correctors”, improve processing or folding of F508del mutant protein^16^. To date, various correctors have been identified; how they function is unknown, although they may act directly on mutant CFTR itself or on proteins in the CFTR interactome. Recently, F508del correctors were grouped into three classes^17^. Class 1 correctors improve the interaction between separate domains of CFTR protein, in particular between the nucleotide-binding domain 1 (NBD1) and the cytosolic loop 4 (CL4). The investigational drug VX-809^18^ belongs to this first group of compounds. Class 2 compounds likely interact with the second nucleotide-binding domain (NBD2). Class 3 correctors, which essentially include chemical chaperones like glycerol, directly stabilize NBD1, a particularly important effect since the F508del mutation is located in this domain^17^. Importantly, it has been shown that the use of a single corrector is not sufficient to promote a marked F508del rescue^17^, suggesting that combinations of correctors will be required to achieve a therapeutic effect.

An alternative way to rescue F508del-CFTR is to modify the proteostasis environment by modulating the function of protein(s) involved in quality control or degradation of CFTR in the ER or plasma membrane^19^,^20^. Various studies have identified proteins that likely function in F508del-CFTR mistrafficking and could thus represent useful drug targets. Among them, the ubiquitin ligase RMA1/RNF5 is particularly relevant as it acts at early stages of CFTR biosynthesis and its loss by gene silencing synergizes with a pharmacological corrector to correct folding defects^21^. Therefore, RNF5 pharmacological inhibition could represent an important strategy to rescue CFTR function in CF patients carrying the F508del allele.

Comparison of RNF5 with other potential therapeutic targets has not been undertaken, particularly in a relevant airway/epithelial background. Here, using functional and biochemical assays, we report that RNF5 silencing results in rescue of F508del-CFTR phenotypes comparable to or better than that obtained by targeting other proteins associated with F508del-CFTR mistrafficking and degradation. As validation of RNF5 suitability *in vivo*, we evaluated the effect of RNF5 knockout in transgenic mice expressing F508del-CFTR. RNF5 knockout F508del-CFTR mice exhibited improved intestinal absorption relative to animals expressing wild-type RNF5, as demonstrated by reduced frequency of animals with severely decreased body weight and reduced fecal excretion of biliary acids. These studies point to potential use of RNF5 as a novel target for F508del-CFTR.

## Results

Using information from the literature and data available in the BIOGRID Protein Interaction Database, we generated a list of proteins that potentially play a key role in F508del-CFTR processing and degradation. We included proteins with known interaction with CFTR, such as RNF5/RMA1, CHIP, DERLIN-1, p97/VCP, and CAL^22^,^23^,^24^,^25^. The list was expanded by adding macromolecules belonging to the sumoylation pathway, a process demonstrated to function in CFTR degradation^26^. We also included proteins identified in gene expression profiles of bronchial epithelial cells treated with F508del correctors^27^. For example, ciclopirox, which acts as a F508del-CFTR corrector in CFBE41o- cells^27^, is an iron-chelating agent and, consequently, inducer of the hypoxia-inducible factor 1 cascade^28^. Consequently, we also included hypoxia-related proteins such as VHL, cullin-2 and the prolyl hydroxylase domain (PHD) proteins EGLN-1, -2 and -3. Procollagen prolyl 4-hydroxylases (P4Hs) were included as a control, given that PHDs likely utilize a mechanism similar to that of P4Hs^29^ and based on the report that P4HA1 interacts with CFTR^30^. Among additional controls, we included members of the anion channels and transporters TMEM16 and SLC26 protein families^31^,^32^. We then generated a specific library of short interfering RNAs (siRNAs) including 3–4 siRNAs per target. CFBE41o- bronchial epithelial cells exhibiting stably co-expressing F508del-CFTR and the halide-sensitive yellow fluorescent protein (HS-YFP) were transfected with each siRNA separately. After 48 hours, F508del-CFTR activity in the plasma membrane was assessed by measuring the rate of HS-YFP quenching caused by iodide influx into cells^33^. As a positive control, we treated cells with the investigational drug corrector VX-809^18^.

**Figure 1:**
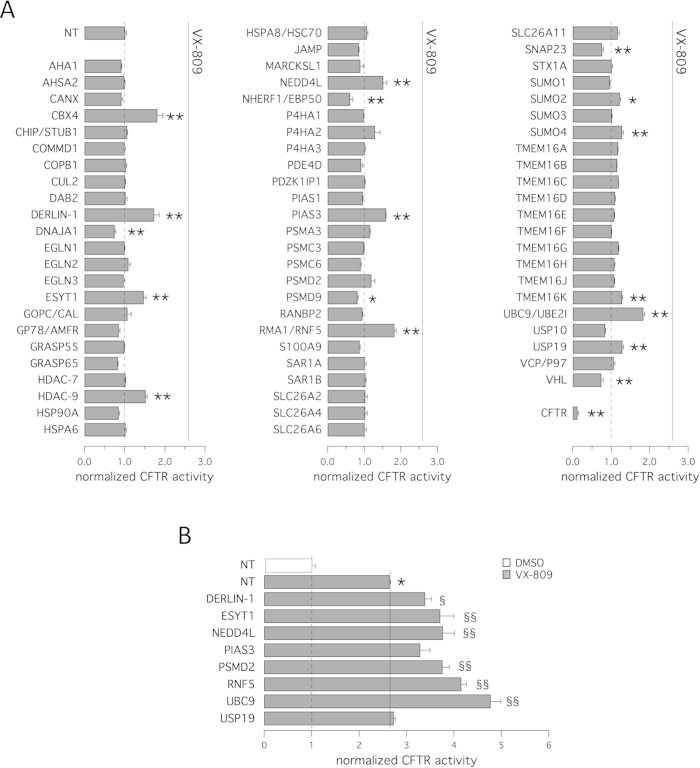
Rescue of F508del-CFTR by gene silencing. The bar graph shows F508del-CFTR activity in CFBE41o- cells based on a YFP assay after transfection with siRNAs (10 nM final concentration) against indicated targets or with control, non-targeting (NT) siRNA (10 nM siRNA in all conditions). The activity measured upon treatments was normalized for the activity detected under control condition (NT-siRNA + DMSO). The assay was carried out 48 hours after transfection. Cells were incubated at 37 °C prior to analysis. Data are expressed as means ± SEM, n = 8. Statistical significance was tested by parametric ANOVA followed by the Dunnet multiple comparisons test (all groups against the control group). Symbols indicate statistical significance versus NT-siRNA: ^**^P < 0.01, ^*^P < 0.05; or versus NT-siRNA + VX-809: ^§§^P < 0.01, ^§^P < 0.05. **A**. Results of siRNA library screening. **B.** Additivity of target silencing with VX-809. Bar graph shows results of combined treatment with vehicle alone (DMSO, white bar) or VX-809 (1 μM; gray bars) of cells transfected with different siRNAs.

Figure 1A summarizes results of the siRNA library screening. Interestingly, we found a significant increase in anion transport based on assessment of HS-YFP quenching following knockdown of some, but not all, targets proposed to function in F508del-CFTR trafficking and degradation. Effectiveness of silencing of specific targets was verified by evaluating target mRNA level using real-time quantitative PCR. For example, silencing of RNF5/RMA1 or DERLIN-1 (silencing achieved 90% and 83%, respectively) elicited a 70–80% increase in F508del-CFTR function over control-transfected cells. RNF5/RMA1 is a ubiquitin E3 ligase responsible for F508del-CFTR degradation at very early steps^21^. DERLIN-1 recognizes misfolded, non-ubiquitylated CFTR to initiate its dislocation and degradation early in the course of CFTR biogenesis^23^. We also observed significant rescue following transfection with siRNAs against NEDD4L, a ubiquitin ligase that regulates ENaC surface expression or internalization downstream of SGK1^34^. Other targets previously associated with F508del-CFTR were not confirmed in our screen, possibly due to poor knockout efficiency. For example, transfection with siRNAs against CHIP, a ubiquitin ligase downstream of RNF5^35^, did not result in rescue of F508del-CFTR trafficking defect. Similar negative results were found for AHA-1, an Hsp90 co-chaperone previously identified as an important target for F508del-CFTR rescue^30^. We also tested histone deacetylase 7 (HDAC-7; silencing achieved 78%), a target of the corrector SAHA^36^, and, as a negative control, we included another histone deacetylase, HDAC-9. Intriguingly, we observed increased anion transport following silencing of HDAC-9 but not HDAC-7.

Among promising targets, we found several proteins functioning in the sumoylation pathway. Indeed, silencing of UBE2I/UBC9 (the primary SUMO E2 conjugating enzyme; silencing achieved 97%) significantly rescued F508del-CFTR protein (80% increase in CFTR activity relative to control cells). Silencing of CBX4, PIAS3 (both SUMO E3 ligases; silencing achieved 87% and 88%, respectively) or SUMO-4 was also effective in increasing anion transport (80%, 60% and 30% increase, respectively). These data confirm the role of sumoylation in F508del-CFTR processing, as described by Frizzell and colleagues^26^. Silencing of PSMD2, a proteasome subunit (silencing achieved 88%), resulted in variable degrees of F508del-CFTR rescue, ranging from no effect to a 60–65% increase in F508del-CFTR activity relative to control-transfected cells.

As an independent control we evaluated the effect of NHERF-1 knockdown. NHERF-1 reportedly stabilizes CFTR at the plasma membrane via direct interaction with the CFTR C-terminus^37^,^38^,^39^. Anti-NHERF-1 siRNAs promoted a significant decrease in F508del-CFTR activity.

Given that combinations of rescue maneuvers (e.g. pairs of correctors or pairs of revertant mutations having different mechanism of action) are required to generate substantial corrective effects on F508del-CFTR activity^17^,^40^,^41^,^42^, we asked whether combining gene silencing with treatment with the corrector VX-809 would result in additive or synergistic effects. Therefore, we transfected CFBE41o- cells with siRNAs and the following day, we treated silenced cells with either vehicle (DMSO) or the corrector VX-809. After additional 24 hours, F508del-CFTR activity in the plasma membrane was measured using the YFP assay. As shown in Fig. 1B, rescue of F508del-CFTR activity by VX-809 treatment was enhanced by loss of specific targets, the most effective being a combination of VX-809 with UBC9 or RNF5 knockdown. Under these conditions, anion transport activity in F508del-CFTR cells markedly increased. Indeed, silencing of UBC9 plus VX-809 caused a 380% increase (relative to cells treated with NT-siRNA plus vehicle), and a 210% increase (relative to cells treated with NT-siRNA plus VX-809) in CFTR-mediated anion transport, while silencing of RNF5 plus VX-809 determined a 310% increase (relative to cells treated with NT-siRNA plus vehicle), and a 140% increase (relative to cells treated with NT-siRNA plus VX-809) in F508del-CFTR activity.

**Figure 2:**
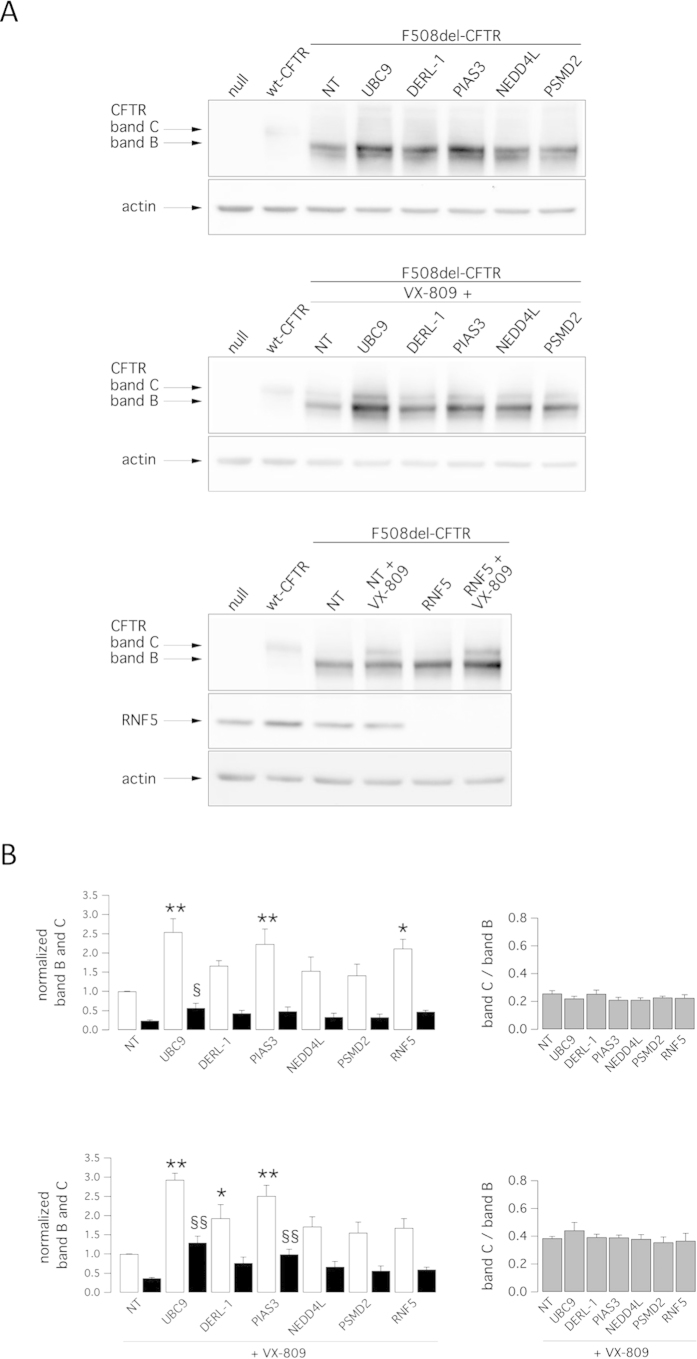
Biochemical analysis of the F508del-CFTR expression pattern. Shown is electrophoretic mobility of F508del-CFTR in CFBE41o- cells after transfection with indicated siRNAs (30 nM final concentration) in combination with treatment with vehicle alone (DMSO) or VX-809 (1 μM). Cells were reverse-transfected with siRNA and cultured for 24 h at 37 °C and then treated with DMSO or VX-809 for an additional 24 h. **A.** Immunoblot detection of CFTR. Arrows indicate complex-glycosylated (band C) and core-glycosylated (band B) forms of CFTR protein. **B.** Quantification of CFTR bands. Left panels: data are expressed as relative abundance of band B (white bars) and band C (black bars), normalized for abundance of band B in cells treated with NT-siRNA + DMSO (top panel) or NT-siRNA + VX-809 (bottom panel). Data are expressed as means ± SEM, n = 3–5 independent experiments. Statistical significance was tested by parametric ANOVA followed by the Dunnet multiple comparisons test (all groups against the control group). Symbols indicate statistical significance versus NT-siRNA band B: ^**^P < 0.01, ^*^P < 0.05; or versus NT-siRNA band C: ^§§^P < 0.01, ^§^P < 0.05. Right panels: graphs reporting the C/B band ratio for data shown in left panels.

**Figure 3:**
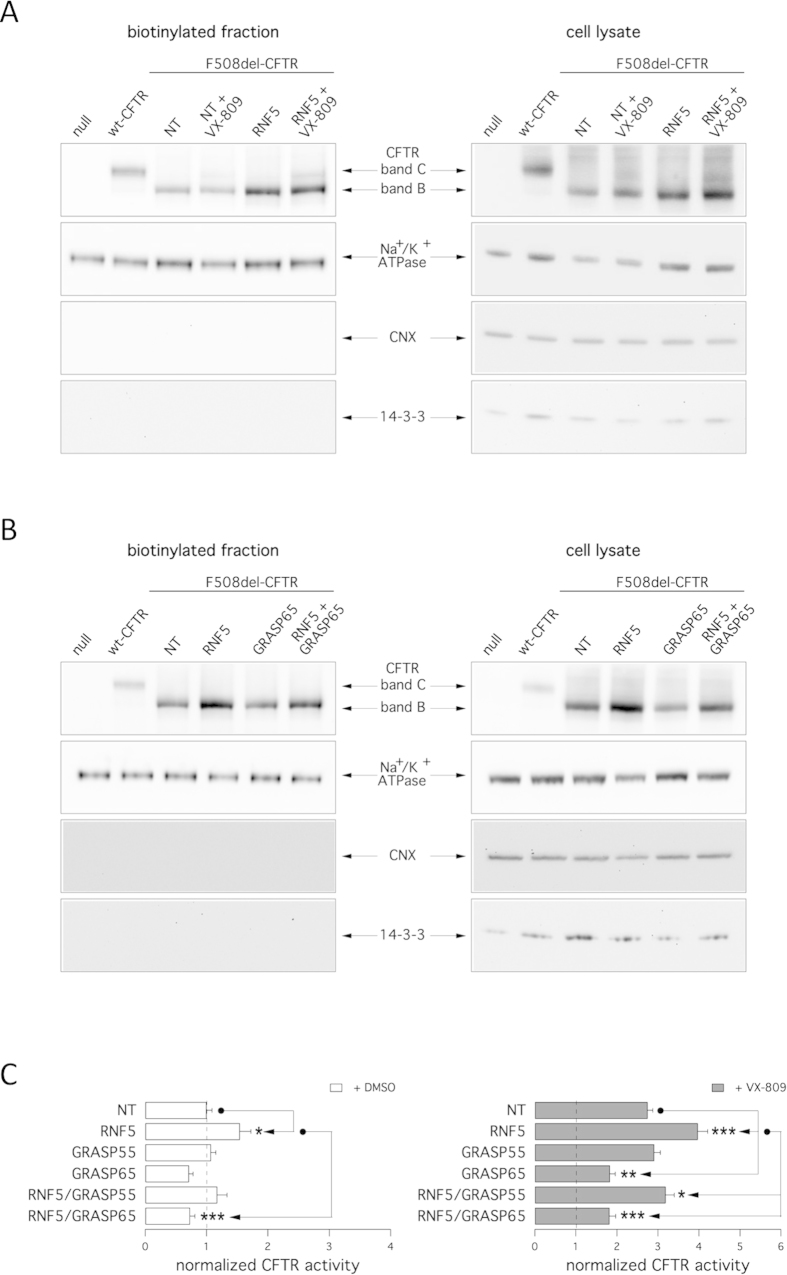
Cell surface expression of complex- and core-glycosylated CFTR. Detection by cell surface biotinylation of F508del-CFTR forms expressed at the plasma membrane. CFBE41o- cells were transfected with indicated siRNAs (30 nM final concentration) and treated with and without VX-809 (1 μM). **A**,**B.** Immunoblot detection of CFTR and control proteins in the biotinylated fraction (left) and in total lysates (right). Arrows indicate the complex-glycosylated form (band C) and core-glycosylated form (band B). Absence of the cytosolic proteins calnexin (CNX) and 14-3-3 in the biotinylated fraction confirms surface protein-specific labeling in each experiment. **C.** The bar graphs show F508del-CFTR activity in CFBE41o- cells (based on a YFP assay) after transfection with indicated siRNAs (10 nM final concentration) combined with treatment with vehicle alone (DMSO; left graph) or VX-809 (1 μM; right graph). The activity measured upon treatments was normalized for the activity detected under control condition (NT-siRNA + DMSO). Data are expressed as means ± SEM, n = 8 per experiment. Reproducibility of results was confirmed by performing three independent experiments. Statistical significance was verified by ANOVA followed by the Tukey test (for multiple comparisons). Symbols indicate statistical significance versus the respective control.

We also assessed rescue phenotypes biochemically by observing the electrophoretic mobility of CFTR protein. On Western blots, CFTR protein is detected as two bands, named B and C, of approximately 150 and 170 kDa, respectively. Band B corresponds to partially glycosylated CFTR residing in the ER. Band C is instead the mature fully processed CFTR that has passed through the Golgi^33^. The prevalent form in cells expressing wild-type CFTR is band C. Cells expressing F508del-CFTR express primarily band B, consistent with severe trafficking defects caused by the mutation (Fig. 2A). To evaluate the effect of target knockdown on CFTR electrophoretic mobility, we transfected CFBE41o- cells with siRNAs and after 24 hours we treated silenced cells with VX-809 (or vehicle). The following day, cells were lysed and lysates were subjected to SDS-page followed by western blotting. Western blot images were analyzed with ImageJ software. For each lane, CFTR bands, analyzed as ROI, were quantified after normalization for actin to account for total protein loading and expressed as relative abundance with respect to band B observed in control cells. Treatment of F508del cells with siRNAs targeting PIAS3 or RNF5 significantly enhanced expression of immature CFTR (band B), while UBC9 knockdown enhanced expression of both immature and mature CFTR without changing the relative ratio of band C to band B (Fig. 2A,B). Incubation of cells for 24 hours with VX-809 was the only treatment that elicited a significant 2-fold increase in C/B band ratio. Combining VX-809 treatment with DERLIN-1 silencing further increased the intensity of band B, while VX-809 treatment combined with either UBC9 or PIAS3 silencing increased both band B and band C intensity relative to cells treated with VX-809 alone without changing the C/B ratio (Fig. 2A,B). Interesting, silencing of RNF5 in combination with VX-809 treatment caused a modest increase in intensity of bands B and C relative to VX-809 treatment alone, although that change was not statistically significant (Fig. 2B). This result contrasts with our functional data showing a strong additive effect of VX-809 combined with RNF5 silencing (Fig. 1B). Therefore, we performed cell surface biotinylation experiments to assess which form of CFTR is expressed at the plasma membrane. In cells expressing wild-type CFTR, the mature form was prevalent (Fig. 3A), while cells expressing F508del-CFTR expressed significant levels of immature CFTR on their surface. Importantly, levels of immature CFTR available for biotinylation were markedly increased following RNF5 knockdown (Fig. 3A). In contrast, treatment with VX-809 only promoted the appearance of mature CFTR. Combined VX-809 plus anti-RNF5 siRNA treatment had an additive effect: the intensity of bands B and C increased (Fig. 3A).

To evaluate mechanisms underlying the effect of RNF5 inhibition on enhanced cell surface expression of mutant CFTR we assessed the potential role of the non-canonical export pathway governed by GRASP family proteins GRASP55 and GRASP65, which are implicated in trafficking of the immature CFTR to the plasma membrane^43^. To do so, we used GRASP55 and GRASP65 knockdown by siRNA molecules to ask whether rescue of F508del-associated phenotypes caused by RNF5 silencing was mediated by the GRASP system. Notably, silencing of GRASP65 (silencing achieved 71%) diminished the amount of immature CFTR (band B) expressed at the membrane as assessed by electrophoretic mobility of biotinylated CFTR protein (Fig. 3B) and markedly decreased RNF5 knockdown-dependent rescue of F508del-CFTR function as assessed by halide transport measurements (Fig. 3C). Interestingly, GRASP65 silencing also decreased basal CFTR activity in control cells, suggesting that basal activity of this pathway in CFBE41o^−^ cells is involved in plasma membrane expression of immature CFTR (Fig. 3B,C). GRASP65 suppression also significantly decreased the extent of CFTR rescue obtained following combined RNF5 silencing and VX-809 treatment (Fig. 2D). GRASP55 silencing (silencing achieved 87%) was less effective (Fig. 3C), indicating that in CFBE41o^−^ cells the primary protein functioning in unconventional F508del-CFTR trafficking is GRASP65.

Our data, obtained in a bronchial epithelial cell line, indicated RNF5 may indeed be an important therapeutic target for rescue of F508del-CFTR. We thus set to confirm our initial observations using a native cell system. Our attempts to use primary cultures of human bronchial epithelial cells did not succeed, largely due to the very low efficiency of target knockdown in these cultures. We thus set to further assess the importance of RNF5 in genetic mouse models, using mice expressing the F508del mutant transgene (Cftr^tm1eur^)^44^,^45^,^46^ and Rnf5^−/−^ mice, all on a C57BL/6J genetic background. RNF5 knockout animals were generated by replacing the first 3 exons of the Rnf5 gene with the Neomycin cassette^47^. Rnf5^−/−^ mice exhibit reduced and delayed activation of ER stress markers, compared with wild-type animals^47^. We then crossed Rnf5^−/−^ mice with transgenic mice bearing the F508del mutation, thereby generating animals that express F508del-CFTR (or wild-type counterpart), with and without RNF5.

**Figure 4:**
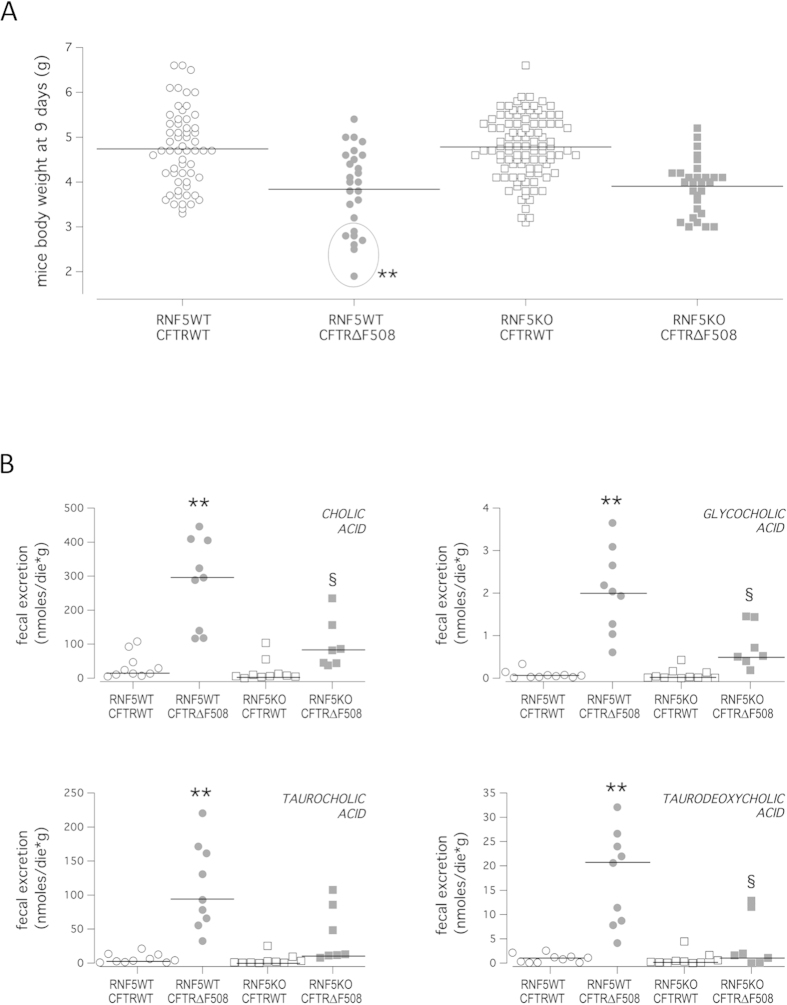
*In vivo* effect of RNF5 suppression in a CF mouse model. **A**. Mouse body weight. Graph shows body weight of 9-day-old littermates (males and females) grouped according to the genotype, as indicated in Methods section. Lines indicate mean values for each group. The quantitative variable “Weight” was dichotomized between mice with RNF5 presence or absence (RNF5WT/CFTR∆F508 and RNF5KO/CFTR∆F508, respectively), and ROC curve anaysis identified a cut-off value of ≤2.9 g (i.e. <3 g). The circle indicates the fraction (26.9% of total) of RNF5WT/CFTR∆F508 mice exhibiting severely reduced body weight (<3 g). Comparison of frequencies of mice with body weight less than 3 grams was performed based on the Fisher’s exact test (as the expected frequencies were less than 5), followed by Bonferroni’s correction. Symbols indicate statistical significance versus the RNF5KO/CFTR∆F508 mice group: ^**^P < 0.01. **B.** Fecal biliary acid excretion. Graphs show the extent of fecal excretion of indicated biliary acids in mice of different genotypes (as for body weight). Lines indicate median values for each group. Littermates are males and females, 15 to 18 weeks of age on a C57BL/6J genetic background. Comparison of values was performed using the non-parametric Kruskal-Wallis ANOVA test, followed by the Mann-Whitney U test and Bonferroni’s correction. Symbols indicate statistical significance versus the RNF5WT/CFTRWT mice group: ^**^P < 0.01; or versus RNF5WT/CFTR∆F508: ^§^P < 0.05.

Lack of functional CFTR in mice mostly results in abnormalities of the gastrointestinal tract, including obstruction and malabsorption, and reduced body weight^46^,^48^,^49^, common characteristics observed in all CF mouse models^46^. On the contrary, the mortality due to CF pathology is more variable^46^. In our strain, the survival-to-maturity rate is >85%^46^. As an important readout of the disease, we analyzed mice body weight. Assessment of body weight was performed in nine-day-old mice. We chose to analyze mouse body weight at 9 days because at earlier stages breastfeeding causes marked weight fluctuations (up to 20% of mouse body weight). In addition, mortality, although low, affects primarily CF pups with the most severe phenotype, hampering long-term studies. Our analysis revealed, as expected, that the mean body weight of wild-type RNF5/homozygous F508del-CFTR (RNF5WT/CFTR∆F508) animals was 25% lower than that of wild-type RNF5/wild-type CFTR (RNF5WT/CFTRWT) mice (Fig. 4A and Table 1). The body weight of RNF5KO/CFTR∆F508 mice was not significantly greater than that of RNF5WT/CFTR∆F508 mice. Homoscedasticity of mouse body weight distributions was verified by using the Hartley F_max_ test, resulting in a significant difference between the variance of RNF5WT/CFTR∆F508 and RNF5KO/CFTR∆F508 groups (P = 0.029). The quantitative variable “Weight” was dichotomized between mice with RNF5 presence or absence (RNF5WT/CFTR∆F508 and RNF5KO/CFTR∆F508, respectively) using the analysis of the ROC curve method; a cut-off value of ≤2.9 g (i.e. <3 g) was obtained. Notably, all the RNF5KO/CFTR∆F508 animals weighed more than 3 g, similar to CFTRWT mice (Table 1). By contrast, about 27% of RNF5WT/CFTR∆F508 animals weighed less than 3 g (circle in Fig. 4A and Table 1). Comparison of frequencies of animals with body weight <3 g between the two groups (RNF5WT/CFTR∆F508 vs. RNF5KO/CFTR∆F508) was performed by means of the Fisher’s Exact test and Bonferroni’s correction. The analysis confirmed that the difference in frequencies is statistically significant (P = 0.00324; P_(B)_ = 0.00647; **). We asked whether there was a difference in the frequency of animals weighting <3 g between RNF5WT/CFTR∆F508 animals having one or two RNF5 wild-type alleles (mice with Rnf5 genotype ^+/−^ or ^+/+^, respectively). We found that the frequencies of animals with body weight <3 g were very similar for the two groups (25.0% and 28.6% for Rnf5 ^+/−^ and ^+/+^, respectively), suggesting that complete RNF5 knockout is required to see the beneficial effect on body weight.

As a second readout of the pathology, we measured the extent of biliary acid (primary, secondary and conjugates) excretion in feces of animals of different genotypes using liquid chromatography-electrospray tandem mass spectrometry. The concentration of primary biliary acids and conjugated biliary acids (bile salts) excreted in feces of RNF5WT/CFTRWT mice was similar to that of RNF5KO/CFTRWT mice (Table 2). As reported previously for FVB Cftr KO and FVB Cftr^tmieur^ mice^48^, RNF5WT/CFTR∆F508 mice exhibited greater excretion of total primary biliary acids and bile salts in stools, than did RNF5WT/CFTRWT (P < 0.01; see Table 2). However, in RNF5KO/CFTR∆F508 mice, we observed decreased excretion of total primary and conjugated bile acids with respect to RNF5WT/CFTR∆F508 mice (Table 2). The most abundant taurine-conjugated biliary salts in mice feces are taurocholic, taurodeoxycholic and taurochenodeoxycholic acids. While levels of the latter did not vary in mice of different genotypes, taurocholic and taurodeoxycholic acid levels showed the same trend as that described for total bile salts excretion (Table 2 and Fig. 4B). Similar trends were observed for the glycine-conjugated biliary salt glycocholic acid, and for the two primary bile acids, cholic acid and chenodeoxycholic acid (Table 2 and Fig. 4B). Such decreases in bile salts excreted in feces demonstrate that *in vivo* RNF5 suppression can improve intestinal absorption in CF mice.

**Figure 5:**
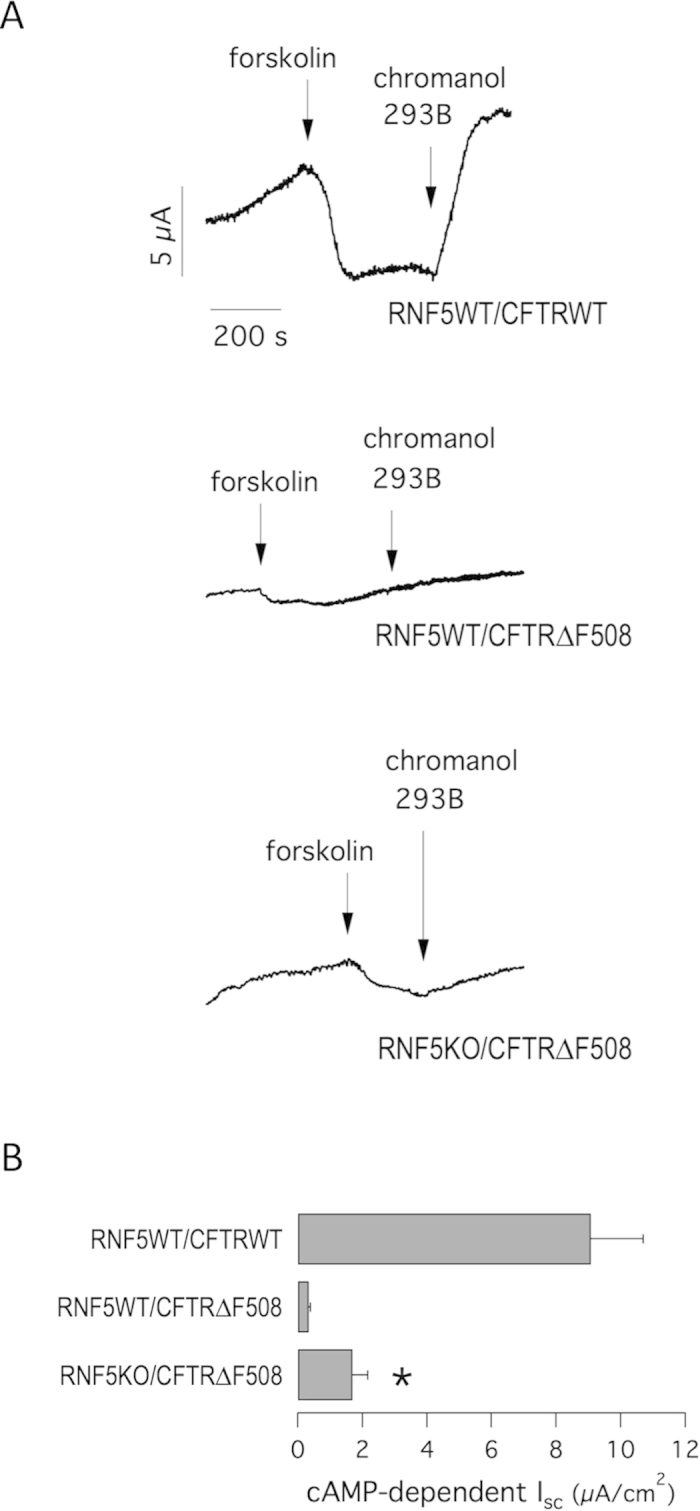
Electrophysiological evaluation of CFTR-mediated activity in mice duodenum. **A**. Representative traces of equivalent short-circuit currents obtained from continuous recordings of transepithelial potential as a function of time obtained from Ussing chamber experiments on mouse duodenum. During recordings tissues were acutely treated with forskolin (10 μM) and chromanol 293B (10 μM). **B**. Summary of equivalent cAMP-dependent ∆I_sc_ in mice of indicated genotypes. Data are expressed as means ± SEM, n = 6–10. Significant differences between data of ∆I_sc_ (RNF5WT/CFTR∆F508 vs. RNF5KO/CFTR∆F508) were calculated using Student’s *t* test. The symbol indicates statistical significance versus the RNF5WT/CFTRWT mice group: *P < 0.05.

We next asked whether increased intestinal absorption observed in RNF5KO/CFTR∆F508 mice is due to increased CFTR activity and sought to characterize ion transport properties of duodenum epithelium from RNF5WT/CFTRWT, RNF5WT/CFTR∆F508 and RNF5KO/CFTR∆F508 mice. To do so, we performed Ussing chamber experiments (Fig. 5A,B). In RNF5WT/CFTRWT animals, addition of the cAMP-elevating agent forskolin to the serosal side of tissue induces a negative shift in transepithelial potential difference (and therefore in the equivalent short-circuit current) reportedly due to cAMP-dependent activation of the apical CFTR and of the basolateral potassium channel KCNQ1/KCNE3, which enables recycling of potassium through the basolateral membrane and maintenance of membrane potential favorable to chloride efflux (Fig. 5A). Subsequent addition of chromanol 293B to the basolateral side of the epithelium to block KCNQ1/KCNE3 channels inhibits anion secretion^50^. As expected, the ∆I_sc_ value for cAMP-induced CFTR-mediated chloride secretion in RNF5WT/CFTR∆F508 mice was minimal and equivalent to <4% the current measured in controls (0.34 ± 0.06 μA vs. 9.1 ± 1 μA in RNF5WT/CFTRWT animals).

In contrast, RNF5KO/CFTR∆F508 mice showed a significant cAMP-induced response (1.7 ± 0.5 μA) with a 5-fold-increase in chloride secretion, equivalent to 18.7% the current measured in controls (Fig. 5B). As for the body weight, we asked whether the ∆I_sc_ value for cAMP-induced CFTR-mediated chloride secretion varied between RNF5WT/CFTR∆F508 animals having one or two RNF5 wild-type alleles (mice with Rnf5 genotype ^+/−^ or ^+/+^, respectively). We found that cAMP-induced responses were very similar for the two groups (0.3 ± 0.1 μA and 0.35 ± 0.08 μA for Rnf5 ^+/−^ and ^+/+^, respectively), suggesting that complete RNF5 knockout is required to see a 5-fold-increase in CFTR-mediated chloride secretion.

These results confirm that phenotypes attributable to CFTR∆F508 can be attenuated *in vivo* by RNF5 ablation, even in the absence of VX-809 treatment.

**Figure 6:**
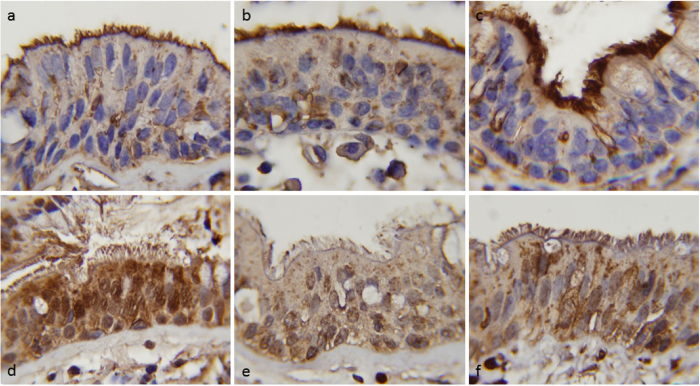
Differential RNF5 expression in human bronchial epithelium of non-CF vs. CF patients. Representative micrographs illustrating immunostain with RNF5 antibody and counterstain with hematoxylin (100X magnification). **a-b-c**: bronchial epithelium of CF patients bearing the F508del mutation; **d-e-f**: bronchial epithelium of control (non-CF) patients (diagnosed as pulmonary fibrosis).

A deregulation of RNF5 expression and localization was observed in certain human myopathies associated with ER impairment, such as inclusion body myositis^47^, but also in some cancers^51^ and in Parkinson’s disease^52^. To assess whether RNF5 protein is differentially expressed/localized in human bronchial epithelia from non-CF vs. CF patients, we performed immunohistochemistry experiments on paraformaldehyde-fixed, paraffin-embedded lung tissues (Fig. 6). Immunostaining with RNF5 antibody revealed that, in CF patients bearing the F508del mutation, a cytoplasmic granular positivity, with paranuclear reinforcement, is present in bronchial epithelial cells, with a marked positivity in the apical membrane beneath the cilia. Differently, in bronchial epithelium of control patients (diagnosed as pulmonary fibrosis), there is a more diffuse and intense cytoplasmic positivity with paranuclear reinforcement with only mild positivity proximal to the cilia (Fig. 6). Semi-quantitative scoring of the signal was assessed on the basis of intensity of RNF5 immunostaining at the apical side and in the cytoplasm and reported in Table 3.

## Discussion

Pharmacological rescue of processing and trafficking defects caused by the CFTR F508del mutation is a critical goal in CF research. This outcome may be achieved by using pharmacological chaperones that directly interact with mutant CFTR to improve its folding and enhance its stability or by blocking activity of proteins that perturb trafficking and promote its premature degradation^19^,^20^. Various proteins are known to affect mutant CFTR maturation/degradation, including proteins of the ubiquitin/proteasome system (RMA1/RNF5, CHIP, DERLIN-1, and NEDD4L), molecular chaperones (AHA-1), proteins of the sumoylation pathway (UBC9), and peripheral scaffolding proteins (NHERF1)^21^,^22^,^23^,^26^,^30^,^34^,^38^,^53^. Here, our goal was to compare the therapeutic relevance of these targets using gene silencing approaches together with functional and biochemical evaluation of F508del rescue, and to evaluate *in vivo* efficacy of target modulation. In particular, we were interested in RNF5, since it acts at early stages of F508del-CFTR biosynthesis. Indeed, early folding defects occurring upon translation of the CFTR nucleotide binding domain 1 (NBD1) are sensed by the RNF5 ubiquitin ligase complex^21^. We report that RNF5 knockdown is particularly effective in rescuing mutant CFTR at the functional level. Although a more effective treatment was UBC9 silencing, *in vivo* suppression of UBC9 is not feasible, due to its pleiotropic effects on the overall sumoylation system. Correspondingly, *ubc9*-deficient mouse embryos die early in development^54^. Intriguingly, our biochemical analysis revealed that RNF5-dependent rescue was based on increased expression and trafficking of the immature form of CFTR protein (band B) to the plasma membrane through a non-canonical route dependent on GRASP protein activity^43^. Specifically, we demonstrate that GRASP65 plays a major role in trafficking of partially glycosylated CFTR to cell surface.

In functional assays we observed an additive/synergistic effect of combining VX-809, a known F508del-CFTR corrector^18^, with RNF5 knockdown. Previously, VX-809 was found to correct F508del-CFTR during its synthesis^55^, hence at a stage when RNF5 is also believed to be involved^21^. Therefore, it was particularly interesting to check if the two interventions had additive effects or not. Our functional analysis reveals that pharmacological correction with VX-809 is additive with RNF5 knockdown. We expected this result to be due to a cooperative effect on F508del-CFTR protein maturation. Indeed, it is possible to hypothesize that blocking mutant CFTR degradation by RNF5 knockdown provides greater substrate availability for VX-809 to act as a pharmacological chaperone. This sequential mechanism should synergistically enhance mutant CFTR maturation. Nonetheless, biochemical analysis revealed that combined drug treatment and RNF5 silencing increased CFTR bands C and B, suggesting that each manipulation affects distinct pools of mutant CFTR. These outcomes raise the intriguing possibility that blocking mutant F508del-CFTR degradation by silencing RNF5 could induce trafficking of the immature form by the GRASP system. Concurrent treatment with VX-809 would impact this process but favor transport of a different CFTR pool to the Golgi and then to the plasma membrane.

Our observations in immortalized bronchial epithelial cells were validated using genetic RNF5KO and CFTR∆F508 animals. Homozygous F508del-CFTR (CFTR∆F508) mice are susceptible to gastrointestinal obstruction and show consistent intestinal malabsorption that dramatically reduces body weight^46^,^48^,^49^, a significantly reduced intestinal cAMP-induced chloride and bicarbonate transport response^46^,^56^, abnormal intestinal mucus secretion^4^,^57^, a higher fecal biliary acids secretion^46^,^48^,^49^. Similar to the human variant, functional correction of murine F508del CFTR by low temperature has been shown in biliary duct cells and intestinal biopsies in this model^45^,^58^. In addition functional correction by proteasome inhibitors in intestinal biopsies^58^ and folding correctors in intestinal organoids has been documented^59^, showing the validity of this model for our purpose. We found that *in vivo* RNF5 suppression ameliorated these defects in CFTR∆F508 model mice. In particular, markedly reduced body weight, seen in approximately 27% of CFTR∆F508 animals, was no longer apparent following RNF5 knockout, although overall mean body weight did not increase. We can speculate that there are individual differences regarding the impact of the relatively increased CFTR activity on body weight in RNF5KO/CFTR∆F508 animals. *In vivo* RNF5 suppression also reduced the extent of fecal biliary acids excretion in these animals. Conjugated bile acids are mainly in their deprotonated form in the duodenum, so that they are more water soluble and able to fulfill their physiologic function of emulsifying fats. Clarke and colleagues demonstrated that in CF mice there is an acidification of the duodenum, resulting from reduced duodenal and pancreatic CFTR-mediated bicarbonate secretion^60^. Lowering of duodenal pH possibly favors conversion of conjugated biliary acids to their protonated form, thus making them less water soluble and more prone to precipitation and excretion in stools. We can speculate that relatively increased CFTR activity seen in RNF5KO/CFTR∆F508 animals is sufficient to limit duodenal acidification, thus avoiding conjugated biliary salt precipitation and improving intestinal absorption.

Our *in vivo* data provide strong validation of biochemical and biophysical analysis performed in cultured cells and clearly indicate the benefits of RNF5 loss. Notably, our *in vivo* assessment was performed in the absence of VX-809. In addition, we could also demonstrate differential RNF5 expression/localization in human bronchial epithelial cells from non-CF and CF patients. Indeed, a deregulation of RNF5 expression and localization was described in different degenerative pathologies including myositis^47^ and Parkinson’s disease^52^. The increase in RNF5 expression seen in these degenerative diseases could be a consequence of ER overload and/or ERAD dysfunction^47^. We can speculate that ER overload caused by F508del-CFTR might result in RNF5 upregulation in a similar way.

These findings provide a strong basis for development of RNF5-targeting molecules that could effectively inhibit its activity *in vivo*. Such inhibitors should ameliorate key pathophysiological phenotypes of patients bearing the F508del mutation. Furthermore, testing of RNF5-targeting molecules in combination with correctors could reveal additional therapeutic benefits. Our work focuses F508del-CFTR; however, it is also important to assess the effect of RNF5-targeting molecules on other CFTR mutants, having trafficking defect, seen in CF patients.

## Methods

### Cell culture

CFBE41o^−^ cells stably expressing F508del-CFTR and the halide-sensitive yellow fluorescent protein (HS-YFP) YFP-H148Q/I152L were generated as previously described^27^. The culture medium was as follows: MEM supplemented with 10% fetal calf serum, 2 mM L-glutamine, 100 U/ml penicillin, and 100 μg/ml streptomycin. For fluorescence assays of CFTR activity, CFBE41o^−^ cells were plated (50,000 cells/well) on clear-bottom 96-well black microplates (Corning Life Sciences, Acton, MA).

### Fluorescence assay for CFTR activity

At the time of the assay, cells were washed with PBS [containing (in mM) 137 NaCl, 2.7 KCl, 8.1 Na_2_HPO_4_, 1.5 KH_2_PO_4_, 1 CaCl_2_, and 0.5 MgCl_2_] and then incubated for 25 min with PBS containing forskolin (20 μM) plus genistein (50 μM). Cells were then transferred to a microplate reader (FluoStar Galaxy; BMG Labtech, Offenburg, Germany) for CFTR activity determination. The plate reader was equipped with high-quality excitation (HQ500/20X: 500 ± 10 nm) and emission (HQ535/30M: 535 ± 15 nm) filters for YFP (Chroma Technology). Each assay consisted of a continuous 14-s fluorescence reading with 2 s before and 12 s after injection of an iodide-containing solution (PBS with Cl^−^ replaced by I^−^; final I^−^ concentration 100 mM). Data were normalized to the initial background-subtracted fluorescence. To determine I^−^ influx rate, the final 11 s of the data for each well were fitted with an exponential function to extrapolate initial slope (dF/dt).

### siRNA library screening

Conditions established for high-throughput siRNA transfection in a 96-well format were as follows: CFBE41o^−^ cells expressing F508del-CFTR and the HS-YFP were reverse transfected with 10 nM (final concentration) siRNAs using lipofectamine 2000 as transfection agent. Twenty-four hours after transfection and plating, the medium was changed and the cells were incubated at 37 °C for additional 24 hours, prior to proceeding with the functional HS-YFP-based assay.

Depending on availability, we utilized siRNA molecules from Dharmacon (pools containing three different duplexes per target) or from Sigma (four different duplexes per target). Results were confirmed using Stealth siRNA molecules from Life Technologies (three duplexes per target). Sequences and/or catalog numbers of siRNAs will be provided upon request. Target silencing was confirmed by evaluating expression of the target mRNA.

### Evaluation of target mRNA level

To evaluate CFBE41o^−^ cell mRNAs, we extracted total RNA using both Trizol reagent (Gibco–BRL) and an RNeasy Mini Kit (Qiagen), both following the manufacturers’ instructions. One μg of spectrophotometer-quantified RNA was retro-transcribed using an iScript RT kit (Biorad). Real-time quantitative PCR (RT-qPCR) was carried out using inventoried Assays-on-Demand provided by Applied Biosystems. The following assays were used: CBX4 (Hs00186344_m1), CFTR (Hs00357011_m1), DERLIN-1 (Hs00919233_m1), GRASP55 (Hs00963853_m1), GRASP65 (Hs00961264_m1), HDAC7 (Hs00248789_m1), PIAS3 (Hs00180666_m1), PSMD2 (Hs01092070_g1), RNF5 (Hs00359834_g1), UBC9 (Hs00163336_m1). Beta2-microglobulin (Hs00187842_m1) served as housekeeping mRNA to normalize transcript abundance. RT-qPCR was performed using an IQ5 Realtime PCR Detection System (BioRad). Cycling conditions were: 3 min hot start at 95 °C, followed by 40 cycles of denaturation at 95 °C for 30 s, and annealing and extension at 60 °C for 30 s. mRNA was quantified using the comparative CT Method. Each sample was run in triplicate, and data were analyzed using IQ5 Optical System software (BioRad). Changes in transcript levels were quantified using the comparative CT Method (Sequence Detection System Chemistry Guide, Applied Biosystems).

### Cell surface biotinylation assay

Parental CFBE41o^−^ cells, CFBE41o^−^ cells expressing native CFTR, or CFBE41o^−^ cells expressing F508del CFTR were seeded on 100 mm dishes and reverse-transfected with 30 nM (final concentration) non-targeting (NT) control siRNAs or siRNAs against selected targets. The day after, cells were incubated with vehicle alone (DMSO) or with VX-809 (1 μM). A cell surface biotinylation assay was performed 24 hr later. Briefly, cells were washed twice with ice-cold PBS and incubated twice with biotin (0.35 mg/ml in PBS) for 25 min each time on a shaker at 4 °C. After three washes in PBS, biotin was quenched with two washes in NH_4_Cl solution (50 mM in PBS, 15 min each) on a shaker at 4 °C. Cells were then washed three times in PBS without Ca^2+^ and Mg^2+^ and then scraped into Lysis Buffer (50 mM Hepes pH 7, 150 mM NaCl, 1% Glycerol, 1% Triton 100X, 1.5 mM MgCl_2_, 5 mM EGTA). Cell lysates were collected in an Eppendorf tube and rocked for 30 min at 4 °C. Nuclei were then pelleted by centrifugation at 10000 rpm at 4 °C for 20 min. Supernatant protein concentration was calculated using the BCA assay (Euroclone) following the manufacturer’s instructions. Then, an aliquot of supernatants corresponding to 600 μg of proteins was precipitated by rotating 6 hr at 4 °C with high capacity streptavidin agarose resin (Thermo Fischer Scientific. Inc), following the manufacturer’s recommendation. The resin was then washed with the following solutions: once with Lysis Buffer, twice with Buffer 1 (150 mM Nacl, 20 mM Tris-HCl, pH 8, 5 mM EDTA, 1% Triton X-100, 0.2% BSA), once with Buffer 3 (150 mM Nacl, 20 mM Tris-HCl, pH 8, 5 mM EDTA, 0.5% Triton X-100), and once with Buffer 4 (50 mM Tris-HCl, pH 8). Biotinylated proteins were eluted from the resin with reducing Sample Buffer 4X and 30 μl of each sample were separated on a 4-to-12% gradient NuPAGE Bis-Tris gel (Life Technologies) and analyzed by Western blotting.

### Western blot

Cells silenced with indicated siRNAs (30 nM final concentration) were grown to confluence on 60-mm diameter dishes and lysed in RIPA buffer containing a complete protease inhibitor (Roche). Lysate protein concentration was calculated using the BCA assay (Euroclone) following the manufacturer’s instructions. Equal amounts of protein (30 μg total per lysate) were separated on 4-to-12% gradient NuPAGE Bis-Tris gels (Life Tecnologies) and analyzed by Western blotting. Proteins were detected using one of the following primary antibodies: mouse monoclonal anti-CFTR antibody (596, Cystic Fibrosis Foundation Therapeutics, University of North Carolina, Chapel Hill); mouse monoclonal anti Na^+^/K^+^ ATPase α1 (cl. C464.6); rabbit monoclonal anti-calnexin antibody (abcam); rabbit polyclonal anti-14-3-3 epsilon antibody (abcam); goat polyclonal anti-actin antibody (cl. I-19, sc-1616, Santa Cruz Biotechnology, Inc) followed by horseradish peroxidase (HRP)-conjugated anti-mouse IgG (Biorad) or HRP-conjugated anti-rabbit IgG (Merk Millipore) or HRP-conjugated anti-goat IgG. Proteins were visualized by chemiluminescence using the SuperSignal West Femto Substrate (Thermo Scientific). Chemiluminescence was monitored using the Molecular Imager ChemiDoc XRS System. Images were analyzed with ImageJ software (National Institutes of Health). CFTR bands were analyzed as ROI, normalized against the actin loading control. Data are presented as means ± SEM of 3–5 independent experiments.

### Animals

RNF5 knockout mice^47^ in C57BL/6J genetic background and recombinant mice bearing the F508del CFTR mutation (Cftr^tm1eur^)^44^,^45^ backcrossed to C57BL/6J^46^ were crossed to establish a colony of double knockout/transgenic mice on a C57BL/6J background and housed in a standard animal facility with solid food (Teklad Global 18% Protein Rodent Diet, code 2018; Harlan Laboratories) and sterilized water ad libitum. Briefly, we crossbred Rnf5^−/−^mice with Cftr^+/F508del^ animals to obtain double heterozygous (Rnf5^+/−^, Cftr^+/F508del^) mice, then, we intercrossed double heterozygous animals. According to the genotype, their offspring was divided into four groups: 1) RNF5WT/CFTRWT (Rnf5^+/+^ or ^+/−^, Cftr^+/+^ or ^+/F508del^) animals; 2) RNF5WT/CFTR∆F508 (Rnf5^+/+^ or ^+/−^, Cftr^F508del/F508del^) animals; 3) RNF5KO/CFTRWT (Rnf5^−/−^, Cftr^+/+^ or ^+/F508del^) animals; and 4) RNF5KO/CFTR∆F508 (Rnf5^−/−^, Cftr^F508del/F508del^) animals. Body weights of female (50%) and male (50%) animals were measured nine days after birth using a 2-digit precision balance. Feces were collected for 96 hr from female (50%) and male (50%) mice at 15–18 weeks of age. For short-circuit current measurements, 8-week-old female (50%) and male (50%) mice were sacrificed in a CO_2_ chamber.

### Mouse genotyping

Genomic DNA was isolated from the mouse tails, using 5’PRIME Kit (Eppendorf), according to the protocol. To detect the F508del-CFTR allele, PCR was carried out in a total volume of 25 μl under the following conditions: 94 °C for 5 min for 1 cycle, followed by 35 cycles of 94 °C for 60 s, 52 °C for 60 s and 72 °C for 30 s, using primers flanking mouse CFTR exon 10 (CF-P580: GGACGCAAAGAAAGGGATAAG; CF-P581: CACAACACTGACACAAGTAGC). The final reaction mixture contained 100 ng of genomic DNA plus Taq Polymerase Master Mix (WWR). Digestion reactions consisting of 10 μl of PCR product, Ssp1 buffer (10X) and 2U of SspI enzyme (Biolabs NewEngland) in a final volume of 15 μl, were incubated for 3 hr at 37 °C and analyzed by 1.5% agarose gel electrophoresis. Digested wild-type PCR products are 308 bp, while the F508del mutation creates a SspI restriction site, which when cleaved in homozygotes produces bands of 184 and 124 bp. Heterozygotes show bands of 308, 184 and 124 bp.

To genotype wild-type and mutant RNF5 alleles, PCR was carried out in 25 μl reactions as follows: 94 °C for 5 min for 1 cycle, followed by 35 cycles of 94 °C for 30 s, 67 °C for 90 s and 72 °C for 30 s. The final reaction mixture contained 100 ng genomic DNA plus Taq Polymerase Master Mix (WWR) and 650 mM betaine (Sigma). Primers used for PCR were as follows: KO-F: GCCAGCTGAAGGTGAGGGACTGGAC, and WT-R: ACACGATGCTGAGGGGAGCTGCAG for the wild-type allele and KO-F and KO-R: TGCGAGGCCAGAGGCCACTTGTGTAGC for the mutated allele. The PCR products were 1700 bp.

### Analysis of fecal biliary acids

Bile salts and acids analytical standards and LC-MS grade solvents were purchased from Sigma Aldrich (Milano, Italy). Instruments, software and UPLC columns were purchased from Waters Inc. (Milford, MA, USA). Bile acids levels in mouse feces were assessed as previously described^61^ with minor modifications. For sample preparation, feces for each mouse were pooled and oven-dried at 45 °C for 96 hr, then 500 mg feces per sample were extracted by homogenization with 4 ml of a 70/30 ethanol/water solution spiked with cholanic acid to a final concentration of 2.5 μM as internal standard (I.S.) and then sonicated 1 hr at room temperature. Samples were then centrifuged 20 min at 9000 g, and 100 μl supernatant was transferred into a 350 μl, 96-well plate and diluted with 100 μl of eluant A. A 4 μl aliquot of supernatant was injected into the LC-MS/MS system for analysis. A 9-points calibration curve of authentic bile acid and salts analytical standard was also prepared in the 10 nM to 100 μM range. Calibrators were processed as described above for the samples and analyzed.

Samples were then analyzed on an Acquity UPLC-TQD LC-MS system. Analytes were separated on a BEH HSS C18 reversed-phase column (2.1 × 100 mm, 1.7 μm particle size). Eluants were: A = water + 0.1% ammonia and B = ACN + 0.1% ammonia. Flow rate was set to 0.5 ml/min with the following gradient profile: 0 to 0.5 min, 5–15% B; 0.5 to 3.5 min, 15–48% B; 3.5 to 4 min, 48–100% B; hold at 100% B for 0.5 min; and then system is reconditioned to 5% B for 1 min. Injection volume was set to 4 μl. As MS parameters, source gas flow and temperature were set to 450 L/hr and 400 °C, respectively, and source temperature was set to 90 °C. Spray voltage (ESI-) was set to 2.0 kV and cone voltage to 50 eV. Analytes were quantified on the basis of their pseudo-MRM traces, setting the quasi molecular ion m/z value (as [M-H]^−^) as both precursor and daughter ion for each MRM transition. Collision energy was set to 5 eV for all transitions. Sample concentration was evaluated on the basis of the analyte to I.S. peak area ratio and compared with standard curve peak areas. Data were quantitatively analyzed used Targetlynx software.

### Intestinal tissue isolation and Ussing chamber measurements

After animals were euthanized, a duodenum segment was excised, rinsed with PBS, and cut open lengthwise through the mesenteric border. Tissue specimens were mounted on a tissue-holding slider (P2303, aperture 0.1 cm^2^) and placed in an Ussing chamber (Physiologic Instruments Inc. San Diego, CA, USA). The solution bathing the apical and basolateral hemi-chambers was continuously gassed with 5% CO_2_ and 95% O_2_ and kept at 37 °C throughout the experiment. That solution consisted of (in mM): 120 NaCl, 25 NaHCO_3_, 3.3 KH_2_PO_4_, 0.8 K_2_HPO_4_, 1.2 MgCl_2_, 1.2 CaCl_2_ and 10 D-Glucose.

The transepithelial potential difference referred to the serosal side was measured using a VCC MC2 amplifier (Physiologic Instrument Inc., San Diego, CA, USA). Current was clamped to 0 μA, and 200 ms pulses of ± 1 μA were passed across the tissue at 1 s intervals. The transepithelial potential difference was recorded using a Lab-Trax-4/16 4-channel data acquisition interface and the Data-Trax data recording and analysis software (World Precision Instruments, Inc. Berlin, Germany).

Measurements of potential difference between the mucosal and serosal compartment (V_te_) allowed calculation of tissue resistance and equivalent short-circuit current (I_sc_). Changes in I_sc_ after treatments, known as ∆I_sc_ values, represent the magnitude of a given flux that was either already present and abolished by a specific drug or a flux that was absent but induced by a given treatment or agonist. The ∆I_sc_ value for cAMP-induced CFTR-mediated chloride secretion was calculated as I_sc_ after addition of 10 μM forskolin minus I_sc_ after subsequent addition of 10 μM chromanol 293B (trans-N-[6-Cyano-3,4-dihydro-3-hydroxy-2,2-dimethyl-2H-1-benzopyran-4-yl]-N-methyl-ethanesulfonamide; Sigma Aldrich)^50^. In this way, anion secretion generates negative ∆I_sc_ values.

### Immunohistochemistry

Immunostaining to detect RNF5 protein was performed using established protocols and reagents^47^,^62^. Briefly, sections from non-CF or CF patients, deriving from paraformaldehyde-fixed, paraffin-embedded lung tissues, were rehydrated by standard methods. Antigen retrieval was performed using Dako target retrieval solution, and endogenous peroxidase was quenched by incubation with 3% hydrogen peroxide for 30 min. Specimens were incubated with anti-RNF5 antibodies (1:200) diluted in Dako antibody diluent overnight at 4 °C. Slides were then washed three times with PBS/Tween-20 and incubated with Dako Labelled Polymer-HRP for 1 h at room temperature. After three washes with PBS/Tween-20, samples were developed with DAB and counterstained with hematoxylin.

### Statistics

Due to the fact that more than 2 groups were to be compared, the Analysis of Variance (ANOVA), followed by a *post-hoc* test was used in order to avoid “multiple comparisons error”. In the case of normally distributed quantitative variables, a parametric ANOVA was performed whereas when the quantitative variables were skewed, the non-parametric ANOVA (Kruskal-Wallis test) was applied. The Kolmogorov-Smirnov test was used to evaluate the assumption of normality.

Statistical significance of the effect of single siRNA treatments on CFTR activity or expression in CFBE41o- cells was tested by parametric one-way analysis of variance (ANOVA) followed by the Dunnet multiple comparisons test (all groups against the control group) as *post-hoc* test. In the case of combination of siRNAs against more than one target, statistical significance was verified by ANOVA followed by the Tukey test (for multiple comparisons) as *post-hoc* test.

Homoscedasticity of mouse body weight distributions was verified by using the Hartley F_max_ test. The quantitative variable “Weight” was dichotomized between mice with RNF5 presence or absence using the analysis of the ROC curve method; a cut-off value of ≤2.9 g (i.e. <3 g) was obtained. Comparison of frequencies of mice with body weight less than 3 grams was performed based on the Fisher’s exact test as the expected frequencies were less than 5, and the post-hoc comparison was made by applying Bonferroni’s correction, and the P value was indicated as P_(B)_.

Values of biliary acids concentration in stools were first tested using Grubb’s test to identify outliers. Comparison of values was then performed using the non-parametric Kruskal-Wallis ANOVA test, followed by the *post-hoc* Mann-Whitney U test with Bonferroni’s correction.

Significant differences between data of short-circuit currents were calculated using Student’s *t* test as the comparison was performed between two groups.

Normally distributed data are expressed as mean ± SEM, while skewed distributed data are expressed as median (min-max), and significances are two-sided. Differences were considered statistically significant when P < 0.05.

### Study approval

The experimental procedures performed during the animal studies were carried out in accordance with the approved guidelines; and were reviewed and approved by the licensing and ethical committee of IRCCS San Martino–IST (Genoa, Italy), and by the Italian Ministry of Health.

## Additional Information

**How to cite this article**: Tomati, V. *et al.* Genetic Inhibition Of The Ubiquitin Ligase Rnf5 Attenuates Phenotypes Associated To F508del Cystic Fibrosis Mutation. *Sci. Rep.* **5**, 12138; doi: 10.1038/srep12138 (2015).

## Figures and Tables

**Table 1:** Body weight of 9-day-old mice with different genotypes.

	**Group 1**	**Group 2**	**Group 3**	**Group 4**
	**RNF5WT/CFTRWT**	**RNF5WT/CFTR∆F508**	**RNF5KO/CFTRWT**	**RNF5KO/CFTR∆F508**
N. Mice	59	26	101	29
Body weight (g): Mean (sem)	4.74 (0.11)	3.84 (0.18)	4.78 (0.07)	3.91 (0.11)
Body weight (g): Median (min–max)	4.7 (3.2–6.6)	4.0 (1.9–5.4)	4.8 (3.1–6.6)	4.0 (3.0–5.2)
N. Mice with body weight <3 g (%)	0 (0%)	7 (26.9%)**	0 (0%)	0 (0%)

Littermates are males and females, of C57BL/6J genetic background. The quantitative variable “Weight” was dichotomized between mice with RNF5 presence or absence (RNF5WT/CFTR∆F508 and RNF5KO/CFTR∆F508, respectively), and ROC curve analysis identified a cut-off value of ≤2.9 g (i.e. <3 g). Comparison of frequencies of mice with body weight less <3 g. was performed based on the Fisher’s exact test (as the expected frequencies were less than 5), followed by Bonferroni’s correction. A symbol indicates a significant difference compared to Group 4; **P < 0.01.

**Table 2:** Fecal biliary acids excretion.

Fecal biliary acids excretion (nmol/day* g)				
	**Group 1**	**Group 2**	**Group 3**	**Group 4**
	**RNF5WT/CFTRWT**	**RNF5WT/CFTR∆F508**	**RNF5KO/CFTRWT**	**RNF5KO/CFTR∆F508**
Cholic acid	19 (5.3–108)	295 (117–446)**	6.7 (0.2–104)	82 (38–235)§
Chenodeoxycholic acid	1.2 (0.3–5.6)	21.8 (6.1–58)**	0.6 (0.01–8.7)	11 (2.5–46)
Total primary biliary acids	21 (5.9–113)	331 (123–468)**	7.1 (0.2–112)	97 (40–281)§
Deoxycholic acid	59 (15–186)	32 (10–132)	31 (0.7–368)	1.4 (1.0–21)§
Lithocholic acid	5.2 (1.4–16)	1.2 (0.4–5.4)*	3.5 (0.1–23)	0.1 (0.1–0.6)§§
Urso+Hyodeoxycholic acid	6.8 (1.6–23)	5.9 (2.1–19)	3.9 (0.1–38)	1.9 (0.6–6.7)§
Total secondary biliary acids	73 (18–221)	39 (13–157)	40 (0.8–429)	3.9 (1.6–29)§
Glycocholic acid	0.1 (0.01–0.3)	2.0 (0.6–3.7)***	0.02 (0.00–0.4)	0.5 (0.2–1.5)§
Taurocholic acid	3.8 (1.0–21)	93 (32–220)**	1.5 (0.02–25)	13 (7.9–108)
Taurochenodeoxycholic acid	1.8 (0.7–11)	1.3 (0.3–9.2)	1.1 (0.02–13)	0.3 (0.02–9.2)
Taurodeoxycholic acid	1.0 (0.1–2.6)	21 (4.1–32)**	0.3 (0.00–4.5)	1.6 (0.00–13)§
Taurolithocholic acid	0.1 (0.1–0.8)	0.2 (0.03–0.7)	0.1 (0.00–1.1)	0.04 (0.01–0.2)
Total conjugated biliary acids	6.9 (1.9–36)	120 (38–250)**	3.1 (0.1–44)	15 (9.6–121)

Values are medians (min–max); n = 7–10. Littermates are males and females, 15–18 wk of age, of C57BL/6J genetic background. Comparison of values was then performed using the non-parametric Kruskal-Wallis ANOVA test, followed by the Mann-Whitney U test and Bonferroni’s correction. Symbols indicate a significant difference versus Group 1; *P < 0.05; **P < 0.01; ***P < 0.001; or versus Group 2; ^§^P < 0.05; ^§§^P < 0.01.

**Table 3:** Semi-quantitative scoring of the signal of RNF5 immunostaining.

**Patient ID (photo ID)**	**Positivity at the apical membrane (beneath cilia)**	**Cytoplasmic positivity and nuclear reinforcement**
CF-1 (a)	3	1
CF-2 (b)	3	1
CF-3 (c)	3	1
control-1 (d)	1	3
control-2 (e)	0	1
control-3 (f)	1	2

Scoring was assessed on the basis of positivity of the immunostaining at the proximal and cytoplasmic level: 0 = absent; 1 = mild, granular; 2 = moderate; 3 = intense, diffuse.
